# Marine Bacterial Polysaccharide EPS11 Inhibits Cancer Cell Growth via Blocking Cell Adhesion and Stimulating Anoikis

**DOI:** 10.3390/md16030085

**Published:** 2018-03-08

**Authors:** Ruobing Cao, Weihua Jin, Yeqi Shan, Ju Wang, Ge Liu, Shan Kuang, Chaomin Sun

**Affiliations:** 1 Key Laboratory of Experimental Marine Biology, Institute of Oceanology, Chinese Academy of Sciences, Qingdao 266071, China; caoruobing15@mails.ucas.edu.cn (R.C.); shanyeqi17@mails.ucas.edu.cn (Y.S.); 18317898332@163.com (J.W.); liug878@163.com (G.L.); kuangshannj@126.com (S.K.); 2 Laboratory for Marine Biology and Biotechnology, Qingdao National Laboratory for Marine Science and Technology, Qingdao 266071, China; 3 Department of Earth Science, University of Chinese Academy of Sciences, Beijing 100049, China; 4 College of Biotechnology and Bioengineering, Zhejiang University of Technology, Hangzhou 310014, China; jinweihua@zjut.edu.cn

**Keywords:** polysaccharide, cancer, filiform structures, βIII-tubulin, anoikis, adhesion

## Abstract

Tumor cells that acquire metastatic potential have developed resistance to anoikis, a cell death process, after detachment from their primary site to the second organ. In this study, we investigated the molecular mechanisms of a novel marine bacterial polysaccharide EPS11 which exerts its cytotoxic effects through affecting cancer cell adhesion and anoikis. Firstly, we found that EPS11 could significantly affect cell proliferation and block cell adhesion in A549 cells. We further demonstrated that the expression of several cell adhesion associated proteins is downregulated and the filiform structures of cancer cells are destroyed after EPS11 treatment. Interestingly, the destruction of filiform structures in A549 cells by EPS11 is in a dose-dependent manner, and the inhibitory tendency is very consistent with that observed in the cell adhesion assay, which confirms that filiform structures play important roles in modulating cell adhesion. Moreover, we showed that EPS11 induces apoptosis of A549 cells through stimulating βIII-tubulin associated anoikis: (i) EPS11 inhibits the expression of βIII-tubulin in both transcription and translation levels; and (ii) EPS11 treatment dramatically decreases the phosphorylation of protein kinase B (PKB or AKT), a critical downstream effector of βIII-tubulin. Importantly, EPS11 evidently inhibits the growth of A549-derived tumor xenografts in vivo. Thus, our results suggest that EPS11 may be a potential candidate for human non-small cell lung carcinoma treatment via blocking filiform structure mediated adhesion and stimulating βIII-tubulin associated anoikis.

## 1. Introduction

Lung cancer, a lethal adult cancer, is one of the leading causes of death worldwide, and the effectiveness of current treatment is severely limited [1]. Non-small cell lung carcinoma (NSCLC) is the most common form which accounts for >80% of all lung cancer cases [2], and the five-year survival rate of NSCLC is about 14% [3]. Moreover, many advanced NSCLC patients have already developed metastasis at the time of diagnosis [4]. However, there is very little progress in the field of blocking tumor metastasis, which remains the primary cause of mortality of lung cancer patients [5]. Therefore, there is a very urgent need to develop more effective therapeutic strategies and novel therapeutic agents that target molecule associated tumor metastasis.

The early steps of metastasis require cancer cells to detach from an extra cellular matrix and migrate away from the primary tumor, and then to survive under anchorage-independent conditions to intravasate into blood or lymphatic circulation [6]. This detachment in most cases leads to the cell death process termed anoikis (detachment-induced apoptosis) [7]. Defects in anoikis lead to the survival of cancer cells during the course of metastasis [6]. Thus, tumor cells that acquire metastatic potential have developed altered mechanisms of cellular adhesion as well as resistance to anoikis [6], which confers a selective advantage for tumor cell invasion and metastasis. Therefore, decreasing cancer cell dissemination by enhancing anoikis appears promising [8].

Adhesion to extra cellular matrix (ECM) is a crucial step for cells’ survival [9], and contacting ECM provides structural support for cells as well as some survival signaling [10]. Promoting detachment of cells from the ECM could enhance anoikis [6]. Therefore, a cell adhesion inhibitor might be a good candidate for inducing cell anoikis [8]. For tumor cells, to successfully proceed through cell adhesion, bundling of actin filaments is essential as it provides rigidity to tumor cells against the compressive forces from the plasma membrane [11]. Filiform structures are the main actin filament-based membrane protrusions which are formed upon remodelling of the actin cytoskeleton beneath the plasma membrane [12]. Filopodium, one of the filiform structures, can be viewed as the sensory organ of tumor cells and plays an important role in detecting and assimilating signals as well as cancer cell adhesion, three-dimensional migration and invasion [13,14]. Therefore, filiform structures are regarded as critical factors for metastatic tumor cells to adhere to the secondary tissues/organs. Metastatic tumor cells are rich in filopodia, and the number determines their invasiveness [15]. Recently, an inhibitor specifically blocking filopodial formation was demonstrated to successfully attenuate breast tumor cell migration and invasion in vitro, and metastasis in vivo [5].

Some cancer cells have the ability to resist anoikis [6], which provides potential in survival and metastasis through hematogenous and lymphatic circulation [16]. It is unclear how cancer cells escape from anoikis, but abnormal expression of certain proteins in tumors has been reported to be one of the key reasons [6]. Notably, βIII-tubulin, one of the critical proteins associated with microtubule assembly, was proven to directly correlate with anoikis sensitivity and metastasis in NSCLC and pancreatic cancer cells [17,18]. There is also some evidence suggesting that βIII-tubulin regulates the protein kinase B (PKB or AKT) signaling pathway, whose activation decreases sensitivity to anoikis [17]. βIII-tubulin blockage in vivo reduced tumor incidence and metastasis [17,18,19], which strongly suggests that silencing its expression may be a potential therapeutic strategy to increase the long-term survival of cancer patients.

Recently, numerous polysaccharides from natural sources have been found to inhibit cancer cell growth and have attracted enormous attention in medical areas [20,21]. However, most of the studied polysaccharides are derived from plants [22], whereas far fewer polysaccharides possessing anticancer properties have been obtained from microbes, especial marine microbes. This is in contrast to the high level of microbial biodiversity in the marine environment, offering a great deal of opportunity for the discovery of novel anticancer agents [22].

In this study, a novel marine bacterial polysaccharide EPS11 was found to significantly affect cell adhesion and induce apoptosis in A549 cells. EPS11 was further demonstrated to inhibit A549 cell growth in vitro and in vivo via destroying filiform structures and stimulating βIII-tubulin associated anoikis. Our results provide direct evidence that EPS11 may be a novel therapeutic agent for NSCLC and further support the roles of βIII-tubulin as an important target in cancer therapy.

## 2. Results

### 2.1. Purification and Identification of Marine Bacterial Polysaccharide EPS11

**Figure 1:**
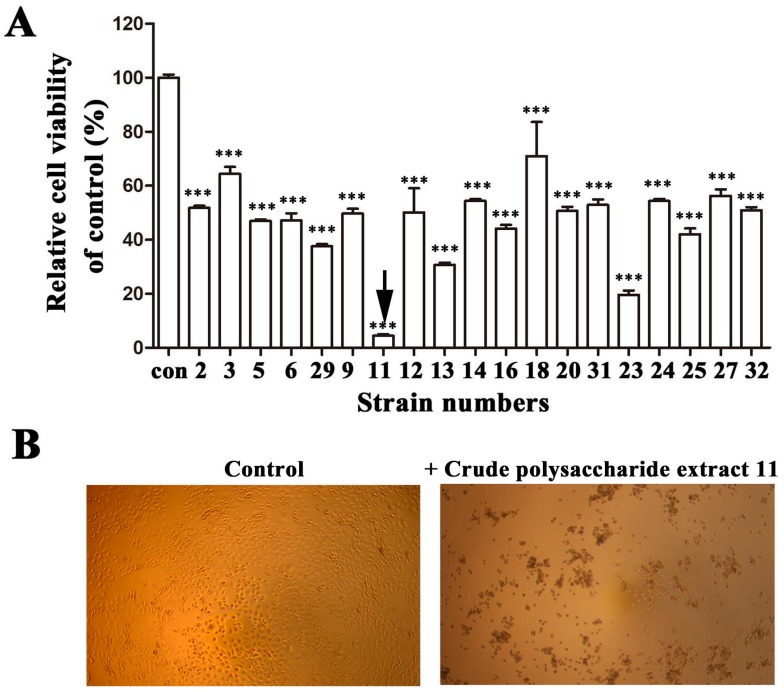

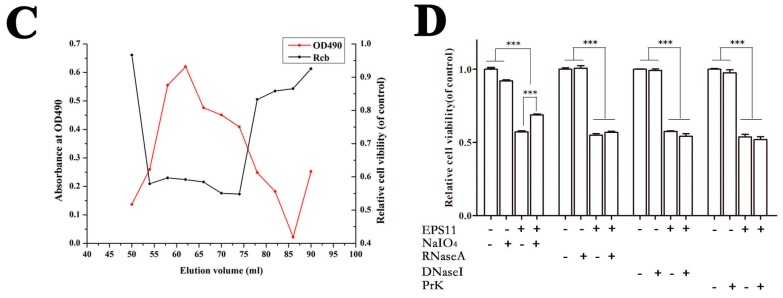
Screening of marine bacterial polysaccharides with cytotoxic activity against A549 cells. (**A**) Cytotoxic effects of crude polysaccharide extracts from different marine bacteria on A549 cells. “Con” represented control group. For the control group, 10 μL sterile water was added into 190 μL cell culture. For the treatment groups, 10 μL crude polysaccharide extract from different bacterium dissolved in sterile water was added into 190 μL cell culture. (**B**) Representative pictures of A549 cells treated without or with crude polysaccharide 11. (**C**) The profiles of the fractions in the gel filtration, which were collected and monitored for the cell proliferation determined at OD570 nm after MTT staining and polysaccharide content determined at OD490 nm after the phenol-sulfuric acid assay. “Rcv” stands for relative cell viability. (**D**) Effects of NaIO_4_, DNase I, RNase A and Proteinase K on the activities of EPS11 inhibiting cell viability in A549 cells. EPS11 (22.5 nM) was respectively treated with proteinase K (100 μg/mL), DNaseI (100 μg/mL), RNaseA (100 μg/mL) or NaIO_4_ (10 mM) for 2 h at 37 °C, then taken to measure the cell viability. Error bars represent standard deviations of three independent experiments. Error bars indicate the standard deviations of 3 measurements. *** *p* < 0.001 versus the control.

In order to obtain potential anticancer marine bacterial polysaccharides, more than 400 crude polysaccharide extracts derived from marine bacteria were screened and evaluated by their abilities to inhibit cancer cells growth using 3-(4,5-Dimethylthiazol-2-yl)-2,5-diphenyltetrazolium bromide (MTT) assay. Among them, strain 11 exhibited the strongest cytotoxic ability. As shown in Figure 1A, the crude polysaccharide extract from strain 11 almost abolished A549 cell growth completely at a dilution of 1:5. Moreover, the cancer cells treated with this crude polysaccharide extract lost adhesion capability and formed evident aggregation (Figure 1B), which indicates that polysaccharide derived from strain 11 might be a strong cytotoxic agent. According to the high homology (99% identity) with marine bacterium *Bacillus* sp. by the 16S ribosomal DNA gene sequencing (Accession no. MG597178), bacterium strain 11 was designated as *Bacillus* sp. 11. 

To elucidate the cytotoxic component from *Bacillus* sp. 11, ethanol precipitation, dialysis, anion exchange and gel filtration were applied to purify the active component from the supernatant of *Bacillus* sp. 11. The relative molecular weight of active component eluted from gel filtration column was estimated to be 22.3 kDa. To verify the polysaccharide characteristics of the active fraction, phenol-sulfuric acid method was used to check the polysaccharide content in the elution fractions. As expected, the cytotoxic activity was positively related to the polysaccharide concentrations (Figure 1C), which suggested the active component might be a polysaccharide. To further confirm the speculation, we used NaIO_4_, RNase A, DNase I and proteinase K to digest the purified active component, respectively. The results showed that treatments with RNase A, DNase I and proteinase K had no effect on the cytotoxic activity of the component. In contrast, treatment with NaIO_4_ reduced the component’s activity significantly (Figure 1D). It is well known that NaIO_4_ is able to hydrolyze polysaccharides by oxidizing the carbon bearing vicinal hydroxyl groups and cleaving the C-C bonds. Therefore, the characteristics of the cytotoxic component indicated that it could be a polysaccharide, which was defined as EPS11 in the following study. Then, high-performance liquid chromatography traces of the polysaccharide hydrolyzate showed monosaccharide components of EPS11 contain mannose, glucosamine, galacturonic acid, glucose and xylose (1:2.58:0.68:0.13:3.09:1.41 in mole ratio).

### 2.2. EPS11 Preferentially Suppressed the Proliferation of Cancer Cells

**Figure 2:**
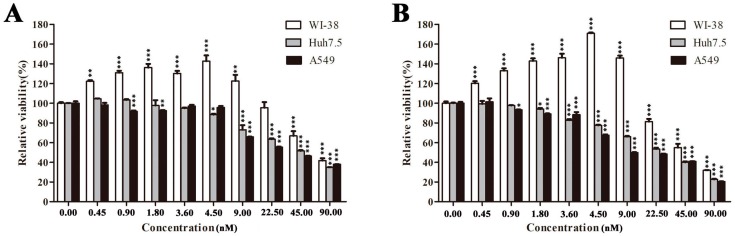
Cytotoxic effects of EPS11 on cell lines WI-38, A549 and Huh7.5. WI-38, A549 and Huh7.5 cells were seeded in 96-well plate overnight, and treated with different concentrations of EPS11 for (**A**) 24 h and (**B**) 48 h. The cell viability was analyzed by MTT assay. Data were presented as means ± SD of three independent experiments (*n* = 3). * *p* < 0.05, ** *p* < 0.01, *** *p* < 0.001.

**Figure 3:**
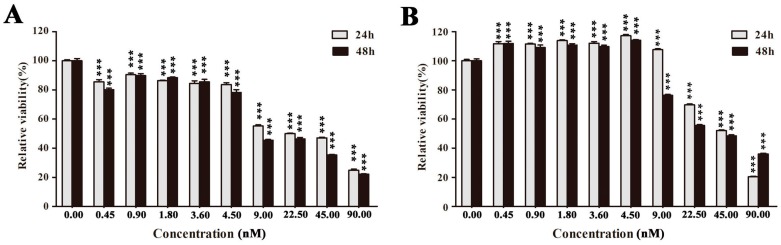
Cytotoxic effects of EPS11 on lung cancer cell lines H1299 (**A**) and H460 (**B**). Cells were seeded in 96-well plate overnight, and treated with different concentrations of EPS11 for 24 h and 48 h, respectively. The cell viability was analyzed by MTT assay. Data were presented as means ± SD of three independent experiments (*n* = 3). *** *p* < 0.001.

To investigate the action mode and therapeutic potential of EPS11, we tested its effects on human cancer and normal cells. Notably, EPS11 preferentially killed cancer cells including human lung cancer cells A549 and HCV-related human liver cancer cells Huh7.5 compared with normal cell line human embryonic lung fibroblasts WI-38. As shown in Figure 2, when the concentration is less than 22.50 nM, EPS11 suppressed the growth of A549 and Huh7.5 cells in time- and dose-dependent manners, while promoting the proliferation of normal cell line WI-38 cells. When the concentration of EPS11 was higher than 22.50 nM, all three above cell lines’ growth was suppressed, while A549 cells were more sensitive than the other two cell lines. The inhibition rate of EPS11 towards A549 cells reached up to 80% after incubation for 48 h at the concentration of 90.00 nM (Figure 2B). The inhibition rates of EPS11 against the other two lung cancer cell lines H1299 and H460 were similar to that of A549 (Figure 3). However, A549 is a frequently used cell line for lung cancer study, thus, we chose A549 as our model to investigate the molecular mechanisms of EPS11.

### 2.3. EPS11 Suppressed Cell Adhesion in A549 Cells

**Figure 4:**
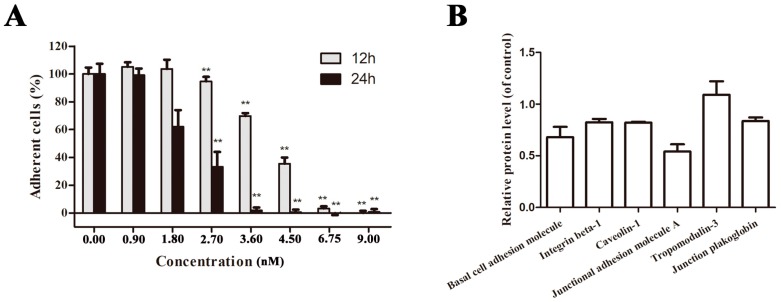
Inhibition of cell adhesion in A549 cells by EPS11. (**A**) Quantification assay of cell adhesion after treatment with different concentrations of EPS11. The data were presented as means + SD of three observation fields in one representative experiment chosen from three independent experiments. * *p* < 0.05, ** *p* < 0.01. A549 cells were treated with or without EPS11 for 24 h. (**B**) Proteomic analysis of the expression of proteins associated with cell adhesion after the treatment with 4.5 nM EPS11 for 24 h.

A549 cell detachment from extra cellular matrix is the most obvious and repeatable effect when treated with either crude extract or purified polysaccharide EPS11. Hence, crystal violet staining was performed to further quantitatively determine the ability of EPS11 to affect cell adhesion in A549 cells. While detached cells were washed off, only adhesive cells can be stained. As shown in Figure 4A, EPS11 significantly decreased the number of adhered cells in time- and dose-dependent manners. When the concentration of EPS11 increased to 9.00 nM, almost all the cells were detached from the extra cellular matrix after 12 h incubation. To further study the effects of EPS11 on cell adhesion in A549 cells, we examined the expression of six reported crucial proteins associated with cancer cell adhesion through proteomic analysis. The six proteins are as follows: human basal cell adhesion molecule, a glycoprotein in cell surface, which is closely related to substrate-adherent [23]; integrin beta-1, a kind of transmembrane proteins, which promotes the formation of small peripheral adhesions and cell protrusions [24]; caveolin-1, a multifunctional membrane protein, whose activation regulates adhesion dynamics [25]; junctional adhesion molecule A, which is known as integral constituents of cellular tight junctions [26]; tropomodulin-3, which acts as a negative regulator of cell adhesion and migration [27]; and junction plakoglobin, which is capable of participating in cell signaling in addition to its role in cell-cell adhesion [28]. The proteomic analysis data showed that levels of expression of five of the six proteins studied was down-regulated (Figure 4B), which is consistent with the result of cell adhesion quantification as shown in Figure 4A. Notably, the only upregulated protein is tropomodulin-3, which was suggested to sequester actin monomers with an affinity similar to its affinity for capping pointed ends and it might be a negative regulator of adhesion [27]. Collectively, we concluded that EPS11 is a strong inhibitor against cell adhesion in A549 cells.

### 2.4. EPS11 Destroyed Filiform Structures and Inhibited Cell Migration in A549 Cells

**Figure 5:**
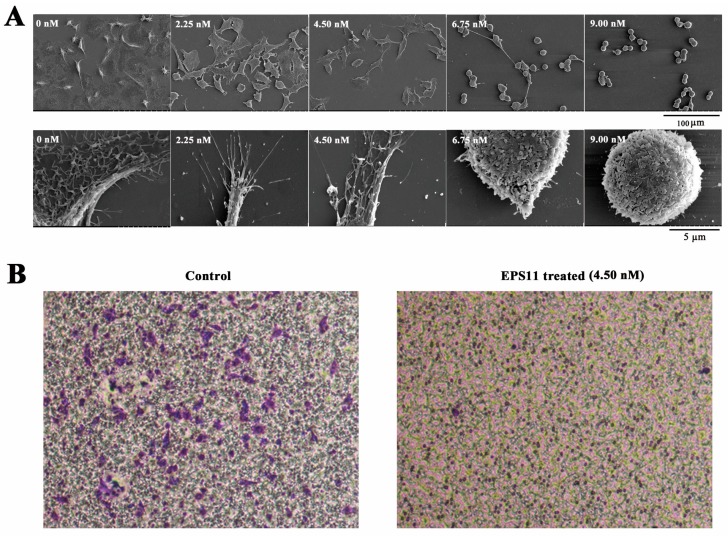
Destroying filiform structures and inhibition of cell migration in A549 cells by EPS11. (**A**) Observation of the filiform structures in A549 cells after treatment with EPS11 via SEM. A549 cells were treated with 0, 2.25, 4.50, 6.75 and 9.00 nM EPS11 for 6 h, respectively. Extracellular structures photomicrographs were observed by SEM under low (upper panels) and high magnification (lower panels). (**B**) Quantitative evaluation of A549 cells migration treated without or with 4.50 nM EPS11 through Transwell assay.

**Figure 6:**
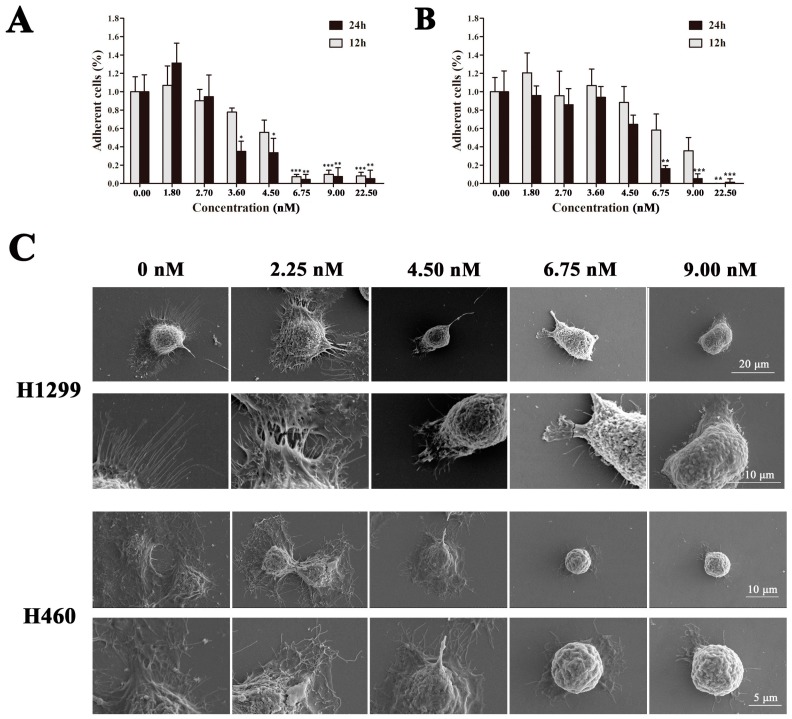
Inhibition of cell adhesion and destroying of filiform structures in lung cancer cell lines H1299 and H460. Quantification assay of cell adhesion in cancer cell lines (**A**) H1299 and (**B**) H460 after treatment with different concentrations of EPS11. The data were presented as means + SD of three observation fields in one representative experiment chosen from three independent experiments. * *p* < 0.05, ** *p* < 0.01, *** *p* < 0.001. H1299 and H460 cells were treated with or without EPS11 for 24 h. (**C**) Observation of the filiform structures in H1299 and H460 cells after the treatment of EPS11 via SEM. H1299 and H460 cells were treated with indicated concentration of EPS11 (0, 2.25, 4.50, 6.75, 9.00 nM) for 10 h.

Given that EPS11 could effectively inhibit cell adhesion in A549 cells, we next sought to check the cells morphology after EPS11 treatment. So, we observed A549 cells with or without EPS11 treatment via scanning electron microscope (SEM). Clearly, A549 cells in control group were multangular with long and multiple filiform structures (Figure 5A, 0 nM treatment), which were used to build association with the extracellular environment. It is noteworthy that the numbers of filiform structures significantly dropped in a dose-dependent manner (Figure 5A). In addition, there were changes in cell shapes where EPS11 treatment led to rounded cells (Figure 5A, 9.00 nM treatment). Specifically, A549 cells lost almost all filiform structures and shifted to a round shape at the concentration of 9.00 nM (Figure 5A, 9.00 nM treatment). The inhibition tendency of filiform structure formation is very consistent with what we observed in the cell adhesion assay (Figure 4A), which confirms that filiform structures play central roles in cell adhesion as described previously [14]. Notably, the cell adhesion and filiform structures in the other two lung cancer cell lines H1299 and H460 were also negatively affected by EPS11 (Figure 6), which was consistent with the results in A549 cells.

Filiform structures are also important factors determining cells migration [5]. EPS11 could effectively attenuate the formation of filiform structures in cancer cells. Therefore, we next asked whether EPS11 could inhibit migration in A549 cells. To address this question, we examined the migration of A549 cells by Transwell assay after treatment with EPS11. As shown in Figure 5B, the migration of A549 cells was significantly suppressed with EPS11 treatment at the concentration of 4.5 nM compared with the untreated group, which strongly indicates that filiform structures are necessary for cell migration in A549 cells.

### 2.5. EPS11 Induced Apoptosis in A549 Cells

**Figure 7:**
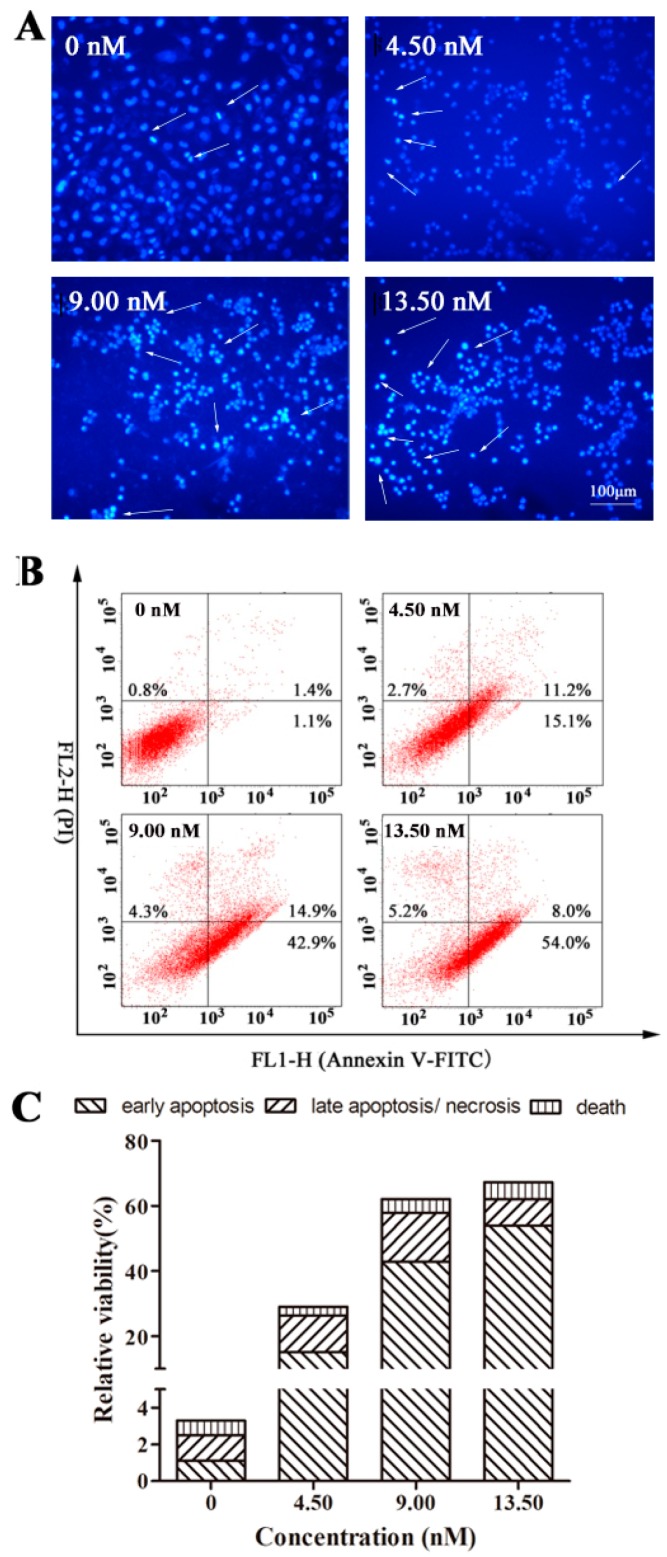
Apoptosis induction in A549 cells by EPS11. (**A**) After treatment with 0, 4.50, 9.00 and 13.50 nM EPS11 for 12 h, A549 cells were stained with Hoechst 33258 solution and visualized by a fluorescence microscopy. White arrows indicated the sublobe, fragment shape, and fringe collection of cell nucleus. (**B**) Representative dot plots of Annexin V/PI staining. After treatment with increasing concentrations of EPS11 (0, 4.50, 9.00 and 13.50 nM) for 24 h, A549 cells were stained with Annexin V-FITC/PI solutions according to the protocol provided by manufacturer, and flow cytometry was used to detect. (**C**) Column bar graph of apoptotic cells. Surviving cells were presented as Annexin V−/PI− (lower left); early apoptotic cells show Annexin V+/PI− (lower right); late apoptotic/necrotic cells were observed as Annexin V+/PI+ (upper right); dead cells were Annexin V−/PI+ (upper left). All experiments were performed three times independently (*n* = 3).

Obviously, EPS11 is a potential inhibitor of cell adhesion (Figure 4 and Figure 6), which suggests that EPS11 might trigger cancer cell anoikis. In order to investigate whether the growth-inhibitory effect is related to the induction of apoptosis, A549 cells were treated with 0, 4.50, 9.00 and 13.50 nM EPS11 for 24 h and the nuclear morphological changes of A549 cells were confirmed by Hoechst 33258 staining (Figure 7A). Compared with the normal nuclear morphology of the control cells, the cells treated with EPS11 presented typical morphological characteristics of apoptosis, including nuclear pyknosis, sublobe, fragment shape, and fringe collection. Further confirmation of apoptosis induced by EPS11 was performed by flow cytometry based on Annexin V-FITC/PI double staining. The results of flow cytometry analysis (Figure 7B,C) showed that the apoptosis of A549 cells were remarkably induced after being treated with EPS11 for 24 h, and treatment of A549 cells with EPS11 resulted in a dose-dependent increase in the numbers of early apoptotic cells, from 1.1% to 54.0%. These data suggested that induction of apoptosis accounted for the growth inhibition of A549 cells treated with EPS11.

### 2.6. EPS11 Downregulated the Expression of βIII-Tubulin and Modulated AKT Activity

**Figure 8:**
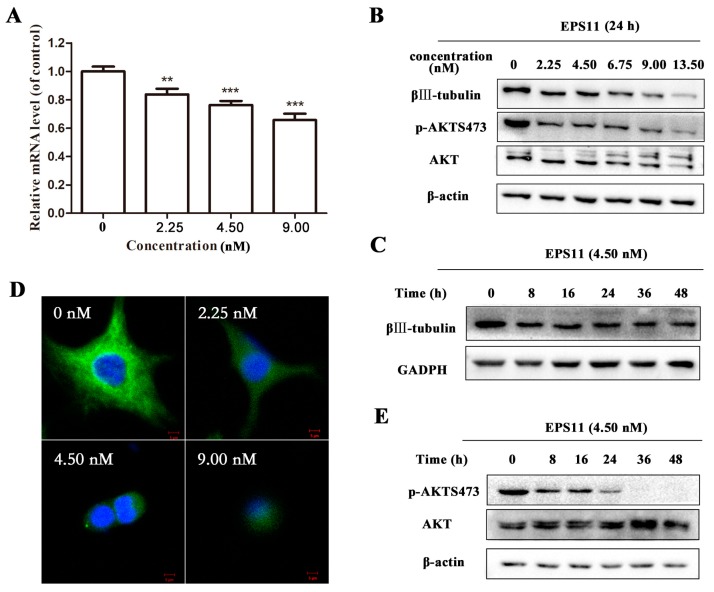
Downregulation of βIII-tubulin expression and AKT phosphorylation by EPS11. (**A**) The mRNA expression levels of βIII-tubulin in A549 cells were downregulated by EPS11. A549 cells were incubated with different concentrations of EPS11 for 24 h, and the mRNA expression of βIII-tubulin was measured by qRT-PCR. β-actin was used as the loading control. * *p* < 0 .05, ** *p* < 0.01, *** *p* < 0.001. (**B**) The protein expression levels of βIII-tubulin in A549 cells were dose-dependently downregulated by EPS11. A549 cells were incubated with different concentrations of EPS11 for 24 h. The expression and phosphorylation levels of AKT were measured by Western blotting. β-actin was used as the loading control. (**C**) The protein expression levels of βIII-tubulin in A549 cells were time-dependently downregulated after treatment with 4.50 nM EPS11 for different times. The protein levels of βIII-tubulin were measured by Western blotting, and GADPH was used as the loading control. (**D**) Inhibition of βIII-tubulin expression in cell. A549 cells were incubated with different concentrations of EPS11 then stained with anti-βIII-tubulin antibody and imaged by fluorescence microscopy. (**E**) Downregulation of AKT phosphorylation by EPS11. A549 cells were incubated with EPS11 at 4.50 nM for different times. β-actin was used as the loading control.

EPS11 induced apoptosis of A549 cells (Figure 7). We next sought to explore the factors associated with this death process after EPS11 treatment. Considering βIII-tubulin is a key factor correlated to anoikis [17], therefore, we explored the expression of βIII-tubulin in both mRNA and protein levels after treatment with EPS11. The result of qRT-PCR showed that EPS11 could dose-dependently decrease the transcription level of βIII-tubulin mRNA (Figure 8A). Consistently, the protein level of βIII-tubulin was also reduced in concentration (Figure 8B) and time-dependent (Figure 8C) manners after treatment with EPS11. To test the inhibitory effect of EPS11 on βIII-tubulin in situ, A549 cells with or without EPS11 treatment were strained with anti-βIII-tubulin antibody and then observed by fluorescence microscope. As shown in Figure 8D, the expression of βIII-tubulin in individual cell was markedly inhibited by EPS11, which is consistent with the results of qRT-PCR and western blot. Collectively, EPS11 could decrease the expression of βIII-tubulin in both transcription and translation levels, suggesting that the induction of apoptosis by EPS11 was at least partly mediated by βIII-tubulin.

AKT is a kind of kinase downstream βIII-tubulin which was reported overactive in tumor cells, and phosphorylation of AKT accounts for un-controlled tumor proliferation and anoikis-resistance generally [29]. So, we further explored whether the AKT signaling was involved in the EPS11-induced apoptosis in A549 cells. As shown in Figure 8E, EPS11 treatment time-dependently downregulated the phosphorylation level of AKT but exerted little effect on AKT expression, which confirmed the suggestion that EPS11 inhibited A549 cells growth via stimulating βIII-tubulin associated anoikis.

### 2.7. EPS11 Attenuated A549 Xenograft Tumor Growth In Vivo

**Figure 9:**
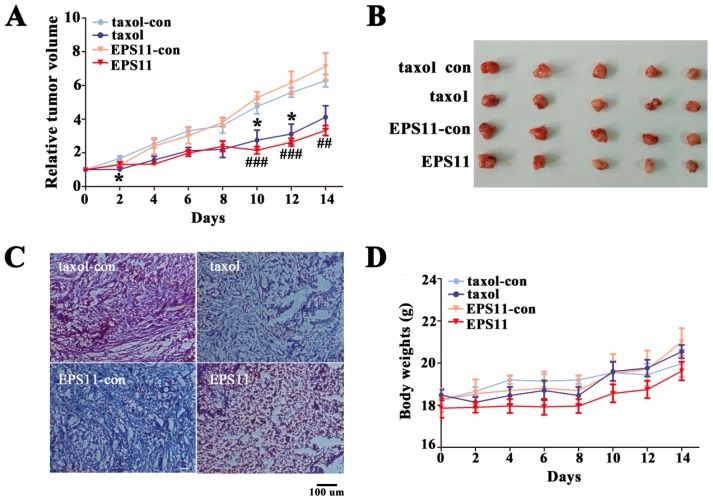
Attenuation of A549 cell xenograft tumor growth in BALB/c-nu mice by EPS11. (**A**) The relative tumor volume (RTV) of each group. * *p* < 0.05 versus control in taxol group; ^##^ *p* < 0.01, ^###^ *p* < 0.001 versus control in EPS11 group. (**B**) Representative images of the A549 xenograft tumors from each group at day 14. Taxol (11.7 μmol/kg d^−1^, intravenous injection) and EPS11 (2.24 μmol/kg d^−1^ on average, intravenous injection) were used for treatment groups. Control groups were treated with the corresponding solvents the same as taxol and EPS11 groups. (**C**) Representative HE (haematoxylin and eosin) staining of the tumor tissues in xenografts in each group as following: taxol-control, taxol, EPS11-control, EPS11. Scale bar = 100 μm. (**D**) EPS11 had little effects on the body weights of treated mice.

To determine whether EPS11 could suppress the growth of tumor in vivo, we evaluated its medicinal effects in BALB/c-nu mice. Taxol is an important clinical chemotherapeutics targeting microtubule, especially β-tubulin ([30]), which was used as positive control for in vivo assay. After 14 days treatment, the relative tumor volumes data suggested EPS11 attenuated A549 xenograft tumors significantly compared with the negative control group, and 5× in mass concentration of the EPS11 behaved similarly to taxol (42.2% versus 46.9%, Figure 9A,B). In addition, after haematoxylin and eosin staining, tumors treated with EPS11 or taxol showed low tissue density compared with the corresponding control group (Figure 9C). No body weight loss and any other abnormalities were observed in the EPS11-treated mice, indicating that EPS11 is a potent and well tolerated compound (Figure 9D).

## 3. Discussion

In recent years, there has been a growing interest in isolating and identifying new microbial polysaccharides possessing anticancer activities [22,31]. Notably, marine bacteria associated with ocean conditions have demonstrated their ability to produce unusual extracellular polymers in an aerobic carbohydrate-based medium [31]. Thus, to find novel microbial polysaccharide with anticancer activities represents an innovative approach to the biotechnological use of under-exploited resources, and it helps in the discovery of more novel therapeutic agents and new therapeutic targets for cancers.

Herein, we report the cytotoxic effects and action mechanisms toward A549 cells by a novel marine bacterial polysaccharide EPS11, including: (1) suppressing the adhesion; (2) destroying filiform structures; (3) inducing anoikis through downregulating the expression of βIII-tubulin and AKT phosphorylation; and (4) attenuating tumor incidence and growth in vivo. Altogether, EPS11 inhibits cancer cell growth via blocking filopodia mediated adhesion and stimulating βIII-tubulin associated anoikis. EPS11 is produced by a marine bacterium *Bacillus* sp. 11 which was isolated from the seamount. Similarly, a marine exopolysaccharide OS-EPS was potent in inhibiting both migration and invasiveness of osteosarcoma cell lines and was very efficient in inhibiting the establishment of lung metastases in vivo [31]. For marine bacteria, we are not clear of the reason for the production of polysaccharides with strong cytotoxic activities against cancer cells, although it probably represents a very ancient defense system due to the direct contact of marine bacteria with high concentrations of microorganisms existing in the ocean environment [22].

Metastasis is one of the major obstacle in cancer therapy and is closely related to high tolerance to first-line chemotherapeutics in NSCLC [32]. It is known that, at a cellular level, metastasis involves several discrete steps including cell adhesion, migration and anoikis resistance, in which tumor cells detach from their primary site, followed by their migration to secondary metastatic sites in distant organs [6]. Adhesion provides mechanical support and survival signals for cells [10], and it is essential for cancer cell growth in both primary and second tumor sites during the course of metastasis [6]. Preventing tumor cells binding to the ECM is an important strategy to inhibit cancer cell spreading and for the induction of anoikis. Thus, the cell adhesion inhibitor has the potential to be developed as an anti-metastasic agent. Actually, it was the phenomenon of evident detachment of cancer cells after EPS11 treatment that attracted our attention when we performed the screening (Figure 1B). Indeed, purified EPS11 could significantly attenuate the adhesion of cancer cells at very low concentrations (Figure 4A and Figure 6). Consistently, the expression of 5 of 6 proteins correlated with cell adhesion was downregulated after EPS11 treatment (Figure 4B). Notably, the five downregulated proteins are also closely related to cell migration, invasion and cell protrusion formation. For example, caveolin-1 was up-regulated in response to the acquisition of anoikis resistance and the up-regulation of caveolin-1 plays important roles in regulation of several cancer cell behaviors including cell growth, anchorage-independent growth, extra cellular matrix adhesion, migration, and invasion, which correlate to cancer cell metastasis [33]. Collectively, our results suggest that the cytotoxic effects of EPS11 are at least partly mediated by suppressing cell adhesion.

Filiform structures are unique organelles widely found in cancer cells, including some primary tumors [34], and they degrade extra cellular matrix, thus promoting invasion and migration of cancer cells [35]. Increased numbers of filiform structures are correlated to increased invasiveness, aggressivity and decreased survival rate [36]. Blocking the formation of filiform structures has been reported to successfully inhibit tumor migration and in vivo metastasis [5]. Besides, filiform structures have less relationship with cells’ viability, which suggests targeting filiform structures may bring little side effects. In our study, filiform structures were markedly destroyed by EPS11 in a dose-dependent manner (Figure 5A and Figure 6C). Interestingly, the concentration damaging filiform structures is consistent with the concentration detaching cells, which indicates that EPS11 is a potential inhibitor of cancer cell adhesion and invasion via destroying the filiform structures. However, the detailed mechanisms of the destruction of filiform structures by EPS11 have yet to be studied in the future.

Cells normally undergo apoptosis, termed anoikis, after they lose contact from neighbouring cells or from their extra cellular matrix [6]. Tumor cells that acquire malignant potential have developed mechanisms to resist anoikis and thereby survive after detachment from their primary site [37]. As a potential inhibitor of cell adhesion and filiform structures’ function, therefore, it is not surprising to find that EPS11 could induce evident anoikis in A549 cells (Figure 7). Recently, overexpression of βIII-tubulin has been proved in many cancer cells, especially the drug-resistant ones [32,38,39], which associates it with cancer cell anoikis resistance. Accordingly, EPS11 inhibited βIII-tubulin expression in both transcription (Figure 8A) and translation (Figure 8B,C) levels, which was further verified in individual cells (Figure 8D). Mechanistically, the PTEN/AKT signaling axis was defined as a critical pathway regulated by βIII-tubulin in NSCLC cells where the phosphorylation level of AKT goes up in proportion to βIII-tubulin expression [17]. As expected, the phosphorylation level of AKT was suppressed with the increase in EPS11 treatment time and showed the same trend as that of βIII-tubulin expression (Figure 8E), which confirms that βIII-tubulin associated anoikis is one of the main reasons for EPS11 killing cancer cells. However, we cannot deny the anoikis-independent apoptosis pathway induced by EPS11 treatment, and it would be interesting to further investigate the action mechanisms of EPS11 against cancer cells.

Moreover, βIII-tubulin has also been associated with drug-resistance in many cases [32,38,39]. Tubulin binding agents (TBAs) bind to β-tubulin subunit and suppress microtubule dynamics, and many first line clinical chemotherapeutics are TBAs, such as paclitaxel, taxanes, and vinorelbine [19]. Overexpression of βIII-tubulin accounts for TBA resistance in different types of cancer, including NSCLC [40,41]. Another defect of this type of tubulin is that it has been proved to restore cancer cells’ sensitive to taxol [30]. As a βIII-tubulin inhibitor, EPS11 have potential synergistic effects with TBAs to combat chemotherapy resistance. In summary, EPS11 inhibits cancer cell growth in vitro via blocking adhesion mediated by filiform structures and inducing βIII-tubulin associated anoikis. As cell adhesion and anoikis sensitivity correlate with cancer metastasis, therefore, EPS11 has potential to be developed as an anti-metastasic agent.

## 4. Materials and Methods

### 4.1. Bacterial Strain Isolation, Identification, and Culture Conditions

Samples used in this study were collected near the Yap Trench during the seamount cruise of the R/V *Kexue* in the tropical Western Pacific in March 2016 (139°3802′ E, 11°44162′ N). The marine bacterial strains used in this study were isolated from the above samples via the dilution method as described previously [42,43], and cultured in modified Zobell 2216E broth (5 g/L tryptone, 1 g/L yeast extract, 1 L filtered seawater, pH adjusted to 7.4–7.6) at 28 °C. The single colonies were further purified in 2216E plates with 1% agar for several rounds as described previously [43]. To phylogenetically classify the marine bacterial strain 11, the 16S rDNA gene sequence was amplified with the universal primers 27F (5′-AGAGTTTGATCCTGGCTCAG-3′) and 1541R (5′-AAGGAGGTGATCCACCC-3′). The 16S rDNA gene sequence of marine bacterial strain 11 was then compared with related sequences in public databases using NCBI-BLAST (http://www.ncbi.nlm.nih.gov/BLAST).

### 4.2. Extraction, Isolation and Purification of Polysaccharides

Extraction of crude polysaccharides from marine bacteria was performed as described previously with minor modifications [44]. Briefly, different bacterial strains were cultured in glass flasks containing 2216E medium with 1% sucrose and incubated at 28 °C under vigorous agitation for 48 h. Cell-free culture supernatant of different strains was collected by centrifugation at 12,000 rpm for 15 min, and precipitated with three volumes of 95% ethanol at 4 °C overnight. The precipitate was collected by centrifugation and dissolved in sterile ddH_2_O, and treated with sevage reagent (chloroform/*n*-butyl alcohol= 5:1; *v*/*v*) twice to remove proteins. Then, the solution was dialyzed against ddH_2_O overnight. The dialyzed fraction was collected for further activity testing.

To obtain the purified polysaccharide, crude polysaccharide EPS11 from 6 L culture treated with sevage reagent was dialyzed against the buffer (50 mM NaCl in 20 mM Tris-HCl, pH 9.0) in 8–14 kDa dialysis bag overnight. The dialyzed fraction was loaded onto a 5 mL HiTrap^TM^ Q HP column (GE Healthcare, Little Chalfont, UK) pre-equilibrated with 50 mM NaCl in 20 mM Tris-HCl (pH 9.0), then eluted with a NaCl gradient (50–500 mM) in the same buffer at 5 mL/min. Active fractions were collected and treated with sevage reagent to remove proteins, and concentrated by ultra-filtration (10-kDa MW cut-off membrane, Millipore, Darmstadt, Germany), then subjected to gel filtration on a Hiload^TM^ 16/600 Superdex^TM^ 200 column (GE Healthcare) pre-equilibrated with 150 mM NaCl in 20 mM Tris-HCl (pH 9.0). The column was eluted with the same buffer and the active fractions were pooled, dialyzed with water and lyophilized for further analysis.

### 4.3. Chemical Analysis of Cytotoxic Component of EPS11

In phenol-sulfuric acid assay, 0.5 mL solutions from each fraction in the gel filtration were mixed with 0.5 mL 5% phenol respectively, then 2.5 mL 95.5% sulfuric acid was added. After mixing and cooling down for 20 min at 20 °C, the polysaccharide content was determined at OD490 nm. For degradation assays, the purified EPS11 with a final concentration of 22.5 nM was incubated with NaIO_4_ (10 mM), RNase A (100 μg/mL), DNase I (100 μg/mL) and Proteinase K (100 μg/mL) for 2 h at 37 °C, respectively. As controls, EPS11 aliquots were incubated in the same conditions in the absence of enzymes or sodium metaperiodate. For each of the above tests, the cytotoxic activities of EPS11 with or without treatment were compared using cell proliferation viability assay described as follows.

### 4.4. Materials for Tissue Culture

Fetal bovine serum (FBS) and RPMI-1640 were purchased from PAN-Biotech (Aidenbach, Germany) and GIBCO (Invitrogen, Grand Island, NY, USA), respectively. Cell apoptosis detection kit was supplied by Key GEN Institute of Biotechnology (Nanjing, China). The enhanced chemiluminescence (ECL) was provided by Pierce (Thermo Scientific, Hudson, NH, USA). Antibodies against β-actin, GAPDH and AKT were purchased from Proteintech, Co., Ltd. (Wuhan, Hubei, China). Antibodies against βIII-tubulin and *p*-AKTS473 were obtained from Cell Signaling Technology (Beverly, MA, USA). Human alveolar basal epithelial cells (A549) were acquired from the Shanghai Institute of Materia Medica, Chinese Academy of Sciences (Shanghai, China). Huh7.5 and WI-38 cell lines were obtained from the American Type Culture Collection. H1299 and H460 cell lines were obtained from the Key Laboratory of Marine Drugs, Ministry of Education, Ocean University of China (Qingdao, Shandong, China). All cell lines were cultured at 37 °C with 5% CO_2_ and 95% air in RPMI-1640 supplemented with 10% FBS.

### 4.5. Cell Proliferation Viability Assay

Viabilities of A549, Huh7.5, WI38, H1299 and H460 were measured by MTT assay. Briefly, different cells (6 × 10^3^/well) were seeded into 96-well plate and cultured at 37 °C for 12 h. Cells were treated with different crude polysaccharides or varying concentrations of EPS11 (0–90.00 nM) for 24 h and 48 h, respectively, and then 30 μL MTT solution (5 mg/mL) was added into each well. After being incubated for 4 h, 100 μL of “Triplex Solution” (10% SDS-5% isobutanol-12 mM HCl) was added to each well for 12 h to dissolve purple crystals of formazan. Absorbance was measured at 570 nm by a multi-detection microplate reader (Infinite M1000 Pro, TECAN, Mannedorf, Switzerland). Relative cell viability was presented as a percentage relative to the control group. All experiments were performed three times.

### 4.6. Cell Adhesion Viability Assay

A549, H1299 and H460 cells (6 × 10^3^/well) were seeded into 96-well plate and cultured at 37 °C for 12 h. Cells were then treated with the varying concentrations of EPS11 (0–9.00 nM) for 12 h and 24 h, respectively. The medium was discarded, and cells were washed three times with PBS. After being fixated with 95% ethanol for 30 min, 80 μL 0.1% crystal violet was added to each well for 20 min. Redundant crystal violet was washed and 100 μL of acetic acid was added to each well with gentle shaking for 10 min to dissolve purple crystals. Absorbance was measured at 590 nm by a multi-detection microplate reader (Infinite M1000 Pro, TECAN, Mannedorf, Switzerland). Relative adhered cells were presented as a percentage relative to the control group. All experiments were performed three times.

### 4.7. Proteomic Analysis

Proteomic analysis was performed by PTM Biolab, Inc. (Hangzhou, Zhejiang, China). Briefly, A549 cells were treated with or without 4.5 nM EPS11 for 24 h and proteins of whole-cell lysates were extracted, separated and digested. The peptides were subjected to Nano electrospray ionization source followed by tandem mass spectrometry (MS/MS) in Q ExactiveTM Plus (Thermo) coupled online to the ultra performance liquid chromatography (UPLC). The resulting MS/MS data were processed using Skyline (v.3.6, Washington, DC, USA). For protein quantitation, a protein was required to contain at least two unique peptides. The protein ratios of treated group/control group were weighted and normalized relative to the median ratio in Mascot.

### 4.8. Scanning Electron Microscope (SEM)

A549, H1299 and H460 cells were seeded onto laminin-coated glass coverslips overnight. Then the cells were incubated with different concentrations of EPS11 (0–9.00 nM) for 6 h, and fixed with 5% glutaraldehyde in PBS and dehydrated of gradient ethanol (30–100%). After treatment with supercritical fluid CO_2_, the coverslips were observed and imaged using SEM (Hitachi S-3400N, Tokyo, Japan).

### 4.9. Transwell Migration Assay

A Transwell Boyden chamber was used to determine the migration of A549 cells. Briefly, the lower compartment contained 0.6 mL of RPMI-1640 medium supplemented with 20% FBS which was added into the lower compartment. A549 cells (5 × 10^5^) were cultured in medium without FBS overnight. Then the cells were resuspended in 100 μL medium containing 1% FBS and different concentrations of EPS11 (0, 4.50 nM), and seeded into the upper compartment of each well. Cells were then incubated for 8 h to allow cell migration through the filter membrane to the lower side of the insert. After being washed with PBS, cells were fixed with 95% ethanol and stained with 0.1% crystal violet. Then, the non-migrated cells on the upper side of the filter were gently removed using cotton swabs and the migrated cells on the lower side of the filter were observed.

### 4.10. Flow Cytometric Analysis of Apoptosis

Apoptosis of A549 induced by EPS11 was detected by flow cytometry (FACS AriaTM II, BD, San Jose, CA, USA) using a commercially available Annexin V-FITC/PI apoptosis detection kit. Briefly, A549 cells (2.5 × 10^6^/well) were seeded into 6-well plate, and treated with different concentrations of EPS11 (0, 4.50, 9.00 and 13.5 nM) for 24 h. The treated cells were harvested and washed with PBS twice. The cell pellets were resuspended in 500 μL of binding buffer, and then 5 μL of Annexin V-FITC and PI was added. After incubation for 10 min at 37 °C in the dark, stained cells were analyzed by flow cytometry.

### 4.11. Hoechst 33258 Staining

After treatment with different concentrations of EPS11 (0, 4.50, 9.00, 13.5 nM), A549 cells were collected and dropped in lysine coated slides. After fixing with 4% paraformaldehyde for 10 min, A549 cells were washed with PBS three times and stained with Hoechst 33258 solution for 10 min in darkness. Thereafter, the cells were observed with Zeiss LSM510 confocal microscopy.

### 4.12. Quantitative Reverse Transcription-PCR (qRT-PCR)

For qRT-PCR, A549 cells treated with different concentrations of EPS11 (0–9.00 nM) were centrifuged at 6000× *g* for 10 min, and total RNAs were extracted using the RNApure Bacteria Kit (DNase I) (CWBio, Beijing, China). Total RNAs were reverse transcribed into cDNA, and the transcriptional levels of different genes were determined by qRT-PCR with Sybr Green Premix Low rox (MDbio, Qingdao, China) and the QuantStudioTM 6 Flex (Thermo Fisher Scientific, Hudson, NH, USA). RNA integrity was assessed by RNA Nano6000 Assay Kit for the Bioanalyzer 2100 system (Agilent Technologies, Santa Clara, CA, USA). Housekeeping gene β-actin was used as an internal reference. The relative gene expression was calculated using the 2^−ΔΔCt^ method with each transcript signal normalized to β-actin. Transcript signals for each treatment were compared to the transcript signals from the control group. Primers tubulin-f (5′-CTGCTCGCAGCTGGAGTGAG-3′) and tubulin-r (5′-CATAAATACTGCAGGAGGGC-3′) were used for amplification of βIII-tubulin encoding gene. Primers actin-f (5′-CACGATGGAGGGGCCGGACTCATC-3′) and actin-r (5′-TAAAGACCTCTATGCCAACACAGT-3′) were used for amplification of actin encoding gene. All qRT-PCR runs were conducted with three biological and three technical replicates.

### 4.13. Western Blot Analysis

A549 cells treated with various concentrations of EPS11 for 24 h were lysed with sample lysis buffer (Sigma-Aldrich, Saint Louis, MO, USA). Then, protein samples were resolved on 8 or 12% SDS-PAGE gels, electro-transferred to nitrocellulose membranes and incubated with primary antibodies (anti-βIII-tubulin, anti-AKT or anti-pAKT) and secondary antibodies, and finally detected by enhanced chemiluminescence. Anti-β-actin and anti-GADPH antibodies were used to normalize protein loading.

### 4.14. Fluorescence Microscopy

After being seeded onto laminin-coated glass coverslips for 12 h, A549 cells were treated with different concentrations of EPS11 (0–9.00 nM) for 24 h. Then cells were fixed with 4% paraformaldehyde for 20 min at 37 °C, permeabilized with 0.1% Triton X-100 for 5 min, and washed three times with PBS. Thereafter, cells were incubated with primary antibody of βIII-tubulin, secondary antibodies and 4′-6-diamidino-2-phenylindole (DAIP). The coverslips were then mounted onto slides and imaged using Zeiss LSM510 confocal microscopy (Jena, Germany).

### 4.15. Xenograft Tumor Model

Four-week-old female athymic (BALB/c) mice were purchased from Beijing Vital River Laboratory Animal Technology Co., Ltd. (Beijing, China). All studies in mice were approved by IOCAS (Institute of Oceanology, Chinese Academy of Sciences, Qingdao, China) Laboratory Animal Care and Ethics Committee in accordance with the animal care and use guidelines. Tumors were established by giving subcutaneous injection of 5 × 10^6^ A549 cells into the left flank of mice. Xenograft mice were administrated i.v. with EPS11 dose at 50 mg/kg (2.24 μmol/kg on average) once every two days, while equivoluminal normal saline was injected as control. Taxol was treated at 10 mg/kg (11.7 μmol/kg) once every two days in the same way as positive control. Taxol was dissolved in 50% anhydrous ethanol and 50% Cremophor EL at 10 mg/mL, to save, and diluted at 0.1 mg/mL to inject. Drug-treatment was started after tumors were palpable and lasted for 14 days. Body weight and tumor volumes were measured every day with a balance or with a vernier caliper. The tumor volume was calculated with the formula: ½ × [length × (width)^2^], and the relative tumor volume (RTV) was calculated with the formula: V*n*/V_0_ (V*n* means the tumor volume at the “*n*” day after administration and V_0_ means the tumor volume before administration). After two weeks of treatment, the mice were euthanized to excise tumors. After weighing, tumors were fixed with 4% paraformaldehyde, and dehydrated on gradient using sucrose solutions in preparation for paraffin section.

### 4.16. Statistical Analysis

All data were presented as means ± SD. Statistical analysis was performed using one-way analysis of variance for comparison of each group by GraphPad Software (5.0, San Diego, CA, USA). Values of *p* < 0.05 were determined to be statistically significant (* *p* < 0.05, ** *p* < 0.01).
